# Histomorphometric Analysis of Callus Formation Stimulated by Axial Dynamisation in a Standardised Ovine Osteotomy Model

**DOI:** 10.1155/2019/4250940

**Published:** 2019-02-12

**Authors:** K. M. Reich, S. Tangl, P. Heimel, S. Lettner, A. Ignatius, L. E. Claes, J. Pfeil, A. Janousek, H. Redl

**Affiliations:** ^1^Karl Donath Laboratory for Hard Tissue and Biomaterial Research, Department of Oral Surgery, University Clinic of Dentistry, Medical University of Vienna, Vienna, Austria; ^2^Austrian Cluster for Tissue Regeneration, Vienna, Austria; ^3^Ludwig Boltzmann Institute for Experimental and Clinical Traumatology, AUVA Research Centre, Vienna, Austria; ^4^Institute of Orthopedic Research and Biomechanics, Center of Musculoskeletal Research Ulm, University Hospital Ulm, Ulm, Baden-Württemberg, Germany; ^5^Department of Orthopaedics and Orthopaedic Surgery, St. Josefs Hospital, Wiesbaden, Germany; ^6^Lorenz Boehler Trauma Center, Vienna, Austria

## Abstract

The cyclic axial dynamisation of a stabilised fracture is intended to promote callus formation and bone healing. Most studies focused on biomechanical properties or the quantity of new bone formation. Far less is known about the quality of newly formed callus tissues, such as tissue distribution and arrangement within the callus. The aim of this current study was to investigate the effect of cyclic, axial dynamisation on the quantity and quality of callus in an established delayed fracture healing model. In 41 sheep transverse osteotomies with a gap size of 3 mm were stabilised with a unilateral external fixator. In 32 of these, fracture ends were axially stimulated with displacement amplitudes of 0.8 mm, 0.4 mm, 0.2 mm, or 0.0 mm, respectively, for six weeks. In the remaining 9 sheep of the control group, an additional external fixator was mounted to achieve almost total rigidity. Animal material originating from a past animal experiment was reanalysed in this study. Histological thin-ground sections were histomorphometrically analysed regarding the histological structure and composition of the defect region. A slight tendency towards an increase in size of total callus area, area of new bone (nB.Ar), and cartilage (Cg.Ar) was detected with increasing displacement amplitudes compared to the control group. At the anterior callus side nB.Ar and Cg.Ar were significantly larger than at the posterior side in all groups independent of treatment. Regarding the quality of callus, areas of very compact bone were predominant in the treatment groups whereas in the control group a slight shift to more porous bone was observed. No difference of callus compactness was observed between the anterior and the posterior side. The established method to assess the local compactness of callus areas is a useful tool to quantitatively determine the spatial distribution of new bone tissue within the callus. The application of this method in combination with biomechanical testing might reveal interesting relations between tissue distribution and bone strength that, with traditional histomorphometry, cannot be identified.

## 1. Introduction

In clinics, most biomechanical and patient-related factors effecting fracture healing are unmodifiable such as the type of (comminuted) fracture, severity of soft tissue injury, blood supply, and also patients' age and pre-existing (inflammatory) diseases [1]. What can be adapted is the rigidity of fracture stabilisation. The use of external fixators for fracture stabilisation allows for the individual adjustment of the mechanical environment which is mainly composed of fracture end stability and mechanical stress particularly applied through weight bearing during the fracture healing process [2]. Rigid devices reduce interfragmentary movement (IFM) and thus mechanical stress on fracture ends, whereas flexible devices enable limited axial loading during weight bearing. With the application of an additional stimulation module the external fixator can stimulate the fracture ends in a controlled manner [3].

The principal idea to mechanically stimulate fractured bone in order to enhance healing is based on Wolff's decisive conclusion on the Mechanotransduction of bone [4]. Due to the ability of bone to sense differences in mechanical strain and to translate this information into signals to stimulate bone remodeling, bone is capable to adapt its mechanical properties according to its present needs. Also, in fracture healing, the biomechanical environment is critical [5, 6]. The external mechanical stimulation of a stabilised fracture site is intended to promote bone healing by simulating loads that naturally occur in healthy bone underweight bearing, for instance. The force, frequency, timing, and duration of externally applied mechanical stimulation via so-termed micromovements can be varied.

In search of optimized stimulation parameters diverse delayed fracture healing models were introduced differing in the choice of animal model, bone region to be studied, osteotomy gap size, type of fixator, magnitude of applied force, timing, frequency, and direction of interfragmentary movement (compression versus distraction)[7, 8]. This variety of studied parameters and the partly diverging results make conclusions on the effect of external mechanical stimulation difficult [7, 9, 10].

It is generally accepted that moderate mechanical stimulation via such applied cyclic axial micromovements is potent to increase callus formation [11–15]. Unlike cyclic loading, static loading seemed to have no effect on bone healing [16]. Shear forces do not impede but seem to prolong the healing process [17]. Large gap size and large interfragmentary strains [9] especially when applied at the wrong time point during the healing process are detrimental to fracture healing [12, 18]. The early phase of fracture healing was shown to be particularly sensitive to mechanical stimulation [12, 19]: some found prolonged healing and an extended chondral phase when the external fixation allowed early IFM [12, 20]. Recently, the focus was put on the modulation of stiffness of fixators during the course of healing [21, 22]. Claes et al. found that primary stability in terms of rigid fixation for at least 3 weeks and following late dynamisation result in superior healing in rats compared to rats with constant flexible or constant rigid fixation [23]. In contrast, the concept of “inverse” or “reverse dynamisation” recommends early flexible fixation during the early healing stages and rigid fixation during the later phases of healing [19, 21, 22, 24]. In these studies, priority was mainly given to clinically relevant parameters such as biomechanical properties (bending stiffness and torsional stiffness) or to the quantity of callus formation in terms of area or volume of newly formed bone [14, 25]. Although the quantity of the callus alone is not a useful predictor for mechanical stability of bone healing [9, 23, 26], far less is known about its quality, that is, the “density” or “compactness” of the newly formed bone tissue and the distribution of tissues within the callus [27, 28].

The aim of this present study was to broaden the current knowledge of the quality and quantity of callus formation by analyzing a well-established delayed fracture healing model in sheep [25, 29, 30] with detailed semiautomatic histomorphometry. Sheep tibiae that have already been employed for two publications [29, 30] were reevaluated for this purpose. Emphasis was put on the arrangement of tissues, that is, the composition and alignment of newly formed bone and cartilage within the callus. Therefore, tibial osteotomies with a gap size of 3 mm were stabilised with unilateral external fixators and stimulated with cyclic axial compression for six weeks. Since best stimulating effects were reported for strains in the range of 0.2 mm to 1 mm [31] we focused in our study on four groups with displacement amplitudes of 0.0 mm, 0.2 mm, 0.4 mm, and 0.8 mm and compared them with a control group equipped with a more rigid two-plane osteotomy fixation device.

We hypothesised that mechanical stimulation with different displacement amplitudes applied daily over a period of 6 weeks has a stimulatory effect not only on the size of callus formation but also on callus distribution and compactness. Based on several studies describing a side-dependent difference in the amount of medial and lateral bone formation within the callus [20, 27, 32], we examined if such regional differences also exist between the anterior and posterior side of the callus. Since mechanical properties of the callus are dependent not only on the quantity but also on the quality of newly formed bone [9, 26, 28, 33], including its porosity, the distribution of areas of low, medium, and high bone density within the callus was analysed.

## 2. Material and Methods

This study reuses sheep tibiae that have already been employed for two publications focusing on the biomechanical and radiological examination of osteogenesis in the cortical bone [29, 30]. Here we addressed new aspects of the same animal model that have not been studied so far and raised novel scientific questions concerning the histological structure and composition of the defect region. While the first two publications concentrated on the question if the method is clinically feasible or not [29, 30], this current study aimed to describe the healing processes at the histological level. New and additional techniques were used for the acquisition of the present data.

Forty-one skeletally mature female merino sheep (average age: 24 months, weighting 50-60 kg) underwent a transverse osteotomy of the left tibia. An osteotomy gap size of 3 mm was produced to simulate a delayed fracture healing model [25]. In all animals osteotomy gaps were laterally stabilised with a custom-made unilateral external fixator comprising a telescoping shaft for controlled axial interfragmentary movement when being unlocked. Sheep were randomly divided into 5 groups: in one group no mechanical stimulation was applied (0.0 mm group). In 3 groups the healing zone of the osteotomy fragments was axially stimulated by means of an attached stimulation microprocessor for 20 min/day with a frequency of 1 Hz and displacement amplitudes of 0.2 mm, 0.4 mm, and 0.8 mm, respectively. Animals of the control group received a unilateral external fixator combined with an additional monotube external fixator (anterolaterally) to increase interfragmentary rigidity and thus disable movement.  Table 1 summarises the treatment groups (including the number of evaluated and excluded animals) and the respective stimulation regimens via the motor-driven external fixator.

The study protocol was approved by the National Commission for Animal Experiments in South Africa (No. 0947, Biocon Research Institute, Pretoria, South Africa). All animal experiments were performed in Pretoria (South Africa) and were in accordance with the institutional guidelines for care and use of animals. Subsequent histological processing was performed in the Karl Donath Laboratory for Hard Tissue and Biomaterial Research, School of Dentistry, Medical University of Vienna (Austria).

### 2.1. Surgery

Detailed surgical protocols, characterisation of the construction, and working mechanisms of the external fixators have been described in greater detail by Wolf et al. [29] and Krischak et al. [30]. In short, a custom-made unilateral external fixator (F. Haas, Künzelsau, Germany) was attached to the exposed lateral tibial diaphysis of all 41 sheep using four 5 mm half-screws (Synthes, Umkrich, Germany). The periosteum was resected 2 mm proximal and distal of the osteotomy line to avoid irritation during sawing. Standardised transverse osteotomy was performed under general anaesthesia using an oscillatory saw and a saw guide, being constantly irrigated with saline solution. Bone fragments were distracted to a gap size of 3 mm and stabilised with the unilateral external fixator. Single knot technique was used to close the superficial layer and skin.

In 9 of the 41 sheep an additional unilateral monotube external fixator (Synthes, Umkrich, Germany) was attached to the anterolateral side of the same tibia with additional four 5 mm half-screws to achieve increased rigidity. These sheep being equipped with a more rigid two-plane fixation configuration are referred to as “*control group*” throughout the text.

During surgery a single dose of ampicillin (1 g) was given for antibiotic prophylaxis. Pin care was performed daily in all animals. Sheep were allowed full weight bearing immediately after surgery.

### 2.2. Stimulation via External Cyclic Compression

In the 32 sheep being exclusively equipped with the custom-made unilateral external fixator, a custom stimulation module was mounted on the telescoping rod of the fixator which was electromechanically driven and controlled by a microprocessor. Animals were randomly divided into 4 treatment groups with different displacement amplitudes of cyclic compression (0.0 mm, 0.2 mm, 0.4 mm, and 0.8 mm). The* 0.0 mm group* received no mechanical stimulation. The other groups, that is,* 0.2 mm group*,* 0.4 mm group*, and* 0.8mm group*, were stimulated 20 minutes per day at a frequency of 1 Hz. Stimulation was started 12 days post-op. To improve legibility, the* 0.0 mm*,* 0.2 mm*,* 0.4 mm*, and* 0.8 mm groups* are referred to as “*treatment groups*” throughout the text.

The rest of the day the telescoping mechanism of the external fixator was locked with screws in order to impede uncontrolled axial interfragmentary movement. Mechanical properties of the external fixators are listed in Table 2. After a healing period of 6 weeks animals were killed with an overdose of pentobarbitone. However, due to deep pin infections and fractures through the screw holes, ten sheep were not able to achieve full weight bearing and thus had to be excluded from the study.

### 2.3. Histology

After sacrificing the animals the external fixators were carefully removed from the dissected tibia. For the preparation of longitudinal undecalcified thin-ground sections bone blocks of the osteotomised tibiae were removed, fixed in neutral-buffered formalin, and embedded in methyl methacrylate (PMMA, Heraeus Kulzer GmbH, Wehrheim, Germany). Longitudinal slices were cut with a band saw, mounted on plastic slides, and ground to a thickness of approximately 100 *μ*m. The polished thin ground sections were finally stained with Paragon.

### 2.4. Histomorphometry

Specimens were photographed with a digital camera (DXM1200; Nikon Cooperation, Tokyo, Japan) mounted on a Microphot FXA microscope (Nikon, Cooperation) at a resolution of 229 pixel/mm. Single images were assembled to create large overview pictures (Lucia G 4.71, Laboratory Imaging Ltd., Praha, Czech Republic) which were further processed in Adobe Photoshop 7.0® (Adobe, San Jose, CA, USA).

The region of interest was defined as the area of the callus 1.5 cm proximal and 1.5 cm distal from the centre of the osteotomy gap, comprising all tissues within the newly formed callus area (new bone, cartilage, and soft/fibrous tissues) but excluding the marrow space surrounding the callus as well as the original proximal and distal autochthonous cortical bone. The lateral margins of the ROI were defined by the maximal extension of the callus.

Based on an algorithm established with Definiens Image Analysis Software® (Definiens AG, Munich, Germany) newly formed bone, cartilage, soft/fibrous tissue, and background were automatically segmented and classified within the region of interest. Falsely classified areas were corrected manually under microscopic control.

Histomorphometric evaluation focused on four aspects:

1. *Total callus area* (*Cl.Ar* in mm^2^) comprising newly formed bone, cartilage, and fibrous tissue encapsulated within the callus present within the given region of interest.
2. Total* area of newly formed bone tissue* (*nB.Ar* in mm^2^) and the total* area of cartilage* (*Cg.Ar* in mm^2^) were measured within the* total callus area*. Besides, new bone volume fraction (*nBV/TV* in %) and cartilage volume fraction (*CgV/TV* in %) were calculated within the* total callus area*, that is, tissue volume (TV).
3. The region of interest was longitudinally divided into the* anterior* and the* posterior callus area*. Newly formed bone area and cartilage area were measured within the* anterior (An.) *and the* posterior (Pt.) callus area*, respectively, [*An.nB.Ar*,* Pt.nB.Ar*,* An.Cg.Ar*,* Pt.Cg.Ar* (in mm^2^)]. Moreover, new bone volume fraction (nBV/TV in %) and cartilage volume fraction (CgV/TV in %) were calculated within these regions.
4. Since the mechanical properties of the callus are dependent not only on the quantity but also on the quality of newly formed bone including its porosity [9, 26], the distribution of new bone within the callus was measured. Therefore, three classes of* new Bone Density (nB.Dn)* were defined by sorting the bone into three brackets based upon their local “bone area density” (not to be confused with bone mineral density as measured by densitometry). Areas where less than 33% were occupied with new bone were defined as regions with* low bone density*, representing bone with high porosity. Areas with 33% to 66% new bone were defined as regions of* medium bone density* areas and areas exceeding the threshold of 66% were classified as* high bone density* regions, representing very compact bone. Morphometrically speaking, the local bone density of each pixel was measured as the bone area fraction within a distance of 0.8 mm around the pixel. Size and distribution of low, medium, and high nB.Dn areas were analysed (Figures 2 and 3).

### 2.5. Statistics

Statistical calculations were conducted using R version 3.4.0 [34]. Mixed models [35] for dependent variables (nB.Ar and Cg.Ar) including a random intercept for animal ID, fixed effects for treatment and location (anterior and posterior), and an interaction term were fitted. For percentages of density areas, an additional level of random effects was included to account for all densities simultaneously. Kenward-Roger approximation for F-tests [36] was used to test for the hypotheses of no effect of several fixed effects.

## 3. Results

**Figure 1:**
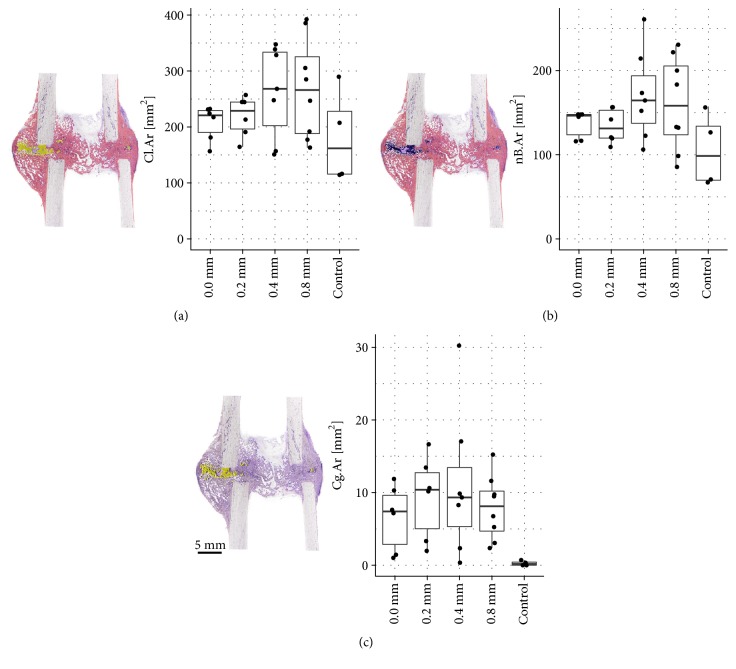
(a)* Total Callus Area (Cl.Ar)*. Total callus area (comprising newly formed bone, cartilage, and fibrous tissue within the ROI) increased with the increase of displacement amplitudes in the treatment groups. Largest Cl.Ar was found in the 0.4 mm group and the 0.8 mm group, smallest callus area in the control group. (b)* New Bone Area (nB.Ar) within the total callus*. Formation of new bone within the total callus area was lowest in the control group. Highest nB.Ar was found in the 0.4 mm group and the 0.8 mm group. (c)* Cartilage Area (Cg.Ar) within the total callus*. Presence of cartilage in the total callus area was very low in all groups. Variation within the groups was very high since cartilage was not present in all specimens. In the treatment groups Cg.Ar was slightly increased compared to the control group.

In total five sheep of the treatment groups and five sheep of the control group had to be excluded from the study as they did not achieve full weight bearing due to deep pin infections and/or fractures through the screw holes. Consequently, callus formation was analysed in six sheep of each, the* 0.0 mm group* and the* 0.2 mm group*, seven sheep of the* 0.4 mm group*, eight sheep of the* 0.8 mm group*, and four sheep of the control group. Because of these unexpected dropouts statistical power of our analysis was very low. Detailed histomorphometric results for the remaining sheep are presented in box plots (Figure 1 and Tables 3 and 4).

### 3.1. Total Callus Area (Cl.Ar)

Total callus area increased with higher displacement amplitudes (Figure 1(a)). The largest Cl.Ar was found in the* 0.8 mm group* (268.5 mm^2^, SD 89.8) and* 0.4 mm group* (262.6 mm^2^, SD 82.9), the smallest callus area in the control group (182.0 mm^2^, SD 83.9). The difference between the groups was statistically not significant (p= 0.342).

### 3.2. nB.Ar in the Total Callus Area

Formation of new bone within the total callus area was lowest in the control group showing a nB.Ar of 105.1 mm^2^ (SD 43.6). In the treatment groups nB.Ar was slightly increased in the* 0.0 mm group* (136.9 mm^2^, SD 16.0) and the* 0.2 mm group* (134.0 mm^2^, SD 20.4) when compared with the control group. Highest new bone formation was found in the* 0.4 mm group* and the* 0.8 mm group* with nB.Ar of 170.5 mm^2^ (SD 53.1) and 160.6 mm^2^ (SD 55.8), respectively. Data are presented in Figure 1(b). The difference between the groups failed to reach statistical significance (p=0.198).

### 3.3. Cg.Ar in the Total Callus Area

Presence of cartilage in the total callus area was very low in all groups (Figure 1(c)). Lowest Cg.Ar was measured in the control group (0.3 mm^2^, SD 0.3). In the treatment groups Cg.Ar was slightly higher ranging from 6.6 mm^2^ (SD 4.5) in the* 0.0 mm group* to 11.1 mm^2^ (SD 10.1) in the* 0.4 mm group*. Variation within the groups was very high since cartilage was not present in all specimens. The difference between the groups was statistically not significant (p=0.206).

### 3.4. nB.Ar in the Anterior and Posterior Callus Area

When dividing the callus axially in an anterior and a posterior region of interest a statistically significant difference in* nB.Ar* between the regions was detected (p=0.001). Detailed results are presented in Table 3.

At the* anterior* side of the callus new bone formation was significantly higher compared to the* posterior* side in all, the control and the treatment groups. Again there was a slight tendency towards an increase of* nB.Ar* in the groups with higher stimulation amplitudes in both regions.

The lowest values for* An.nB.Ar* and* Pt.nB.Ar* were found in the control group (*An.nB.Ar* 70.7 mm^2^, SD 44.0 and* Pt.nB.Ar* 34.4 mm^2^, SD 9.1). Highest* An.nB.Ar *was measured in the* 0.8 mm group* (103.8 mm^2^, SD 36.4) and the* 0.4 mm group* (111.7 mm^2^, SD 38.4). In contrast,* Pt.nB.Ar *was highest in the* 0.2 mm group* (63.1 mm^2^, SD 25.4). The interaction between region and treatment group was not statistically significant (p=0.403).

### 3.5. Cg.Ar in the Anterior and Posterior Callus Area

*Cg.Ar* was significantly higher in the* anterior* callus side compared to the* posterior *callus side (p=0.029).* Anterior Cg.Ar* was found to be smallest in the control group with 0.3 mm^2^ (SD 0.3). Highest* An.Cg.Ar* was found in the* 0.4 mm group* (8.7 mm^2^, SD 9.3). Posteriorly, no cartilage was present in any animal of the control group. In the* 0.2 mm group*,* Pt.Cg.Ar* was highest with 3.5 mm^2^ (SD1.9). In the other groups* Pt.Cg.Ar* was very close ranging from 1.3 mm^2^ (SD 2.4) in the* 0.0 mm group* to 2.4 mm^2^ (SD 3.3) and 2.4 mm^2^ (SD 2.1) in the* 0.4 mm* and* 0.8 mm group*, respectively. See Table 3. The interaction between region and treatment group was not statistically significant (p=0.536).

### 3.6. nBV/TV and CgV/TV

Although the absolute size of the callus area was significantly different between the anterior and the posterior side, the volume fraction of newly formed bone within these given regions was very similar (p=0.421). Detailed histomorphometric results for total nBV/TV and total CgV/TV as well as for the anterior and posterior volume fractions are presented in Table 4.

The parameter nBV/TV, however, only reflects the overall density of the newly formed bone within the callus area. In order to gather information about the actual distribution of the new bone within the callus area, the new parameter* new Bone Density* (nB.Dn) was introduced as follows.

### 3.7. Size of Different Bone Density Areas (nB.Dn)

**Figure 2:**
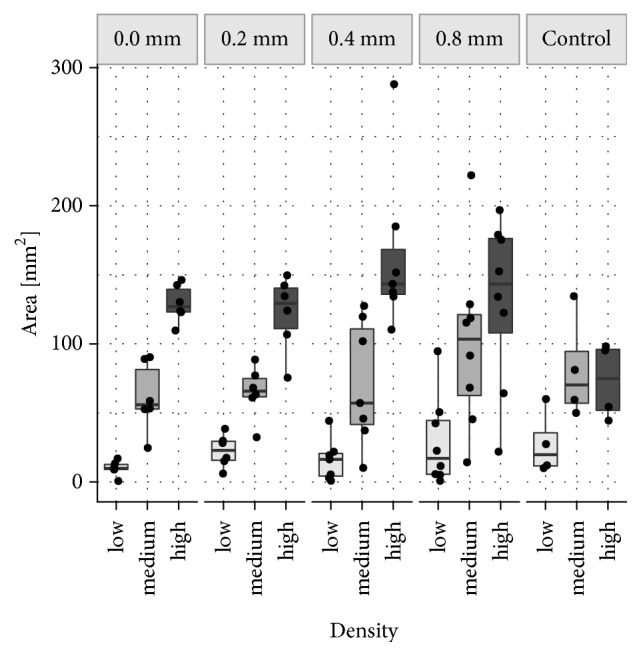
*Size of Different Bone Density Areas (nB.Dn)*. Mineralised callus areas were classified as* low nB.Dn* (areas less than 33% new bone),* medium nB.Dn *(areas with 33 to 66% new bone), and* high nB.Dn* (areas exceeding 66% new bone). In this box plot diagram, the average size of low, medium, and high bone density areas is depicted for the* 0.0 mm*,* 0.2 mm*,* 0.4 mm,* and* 0.8 mm group* as well as for the* control group*.

**Figure 3:**
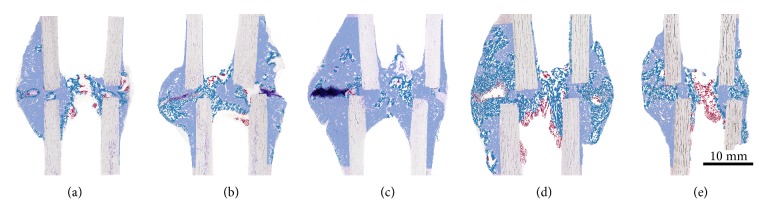
*Areas of Different Bone Densities (nB.Dn) within the Callus*. Histological images were semiautomatically segmented and areas of different bone densities were colour encoded. Mineralised callus areas with less than 33% new bone were defined as regions with* low nB.Dn (red)*, areas with 33 to 66% new bone as regions of* medium nB.Dn (turquoise),* and areas exceeding 66% were classified as* high nB.Dn (light blue)*. For better visualisation, histological images are superimposed by the respective false colour images. The distribution of areas with different bone densities over the total callus area was very similar between the groups and showed a consistent inherent pattern. Besides, treatment groups (a-d) show a very similar proportional distribution: a relatively small area of* low nB.Dn* (5-10%), approximately 30%* medium nB.Dn*, and 50-65%* high nB.Dn*. In the control group (e), a shift to less dense bone was observed: the upwards trend observed in the treatment groups levelled off at the medium density level in the control group (approximately 45%* medium nB.Dn*). Areas with* high nB.Dn* were less common (approximately 42%) as compared to the treatment groups. (a) 0.0 mm group, (b) 0.2 mm group, (c) 0.4 mm group, (d) 0.8 mm group, and (e) control group. Anterior side is on the left and posterior side is on the right.

Differences in the size of different bone density areas are presented in Figure 2. Their distribution within the ROI is illustrated in Figures 3(a)–3(e).

Areas of* low bone density (low nB.Dn)* were defined as callus areas of high porosity, that is, callus areas comprising less than 33% newly formed bone.* Low bone density* areas were very rare in all groups. In the treatment groups* low nB.Dn* areas ranged from 10.0 mm^2^ (SD 5.5) in the* 0.0 mm group* to 29.2 mm^2^ (SD 32.1) in the* 0.8 mm group*. In the control group,* low bone density* areas had a size of 27.4 mm^2^ (SD 23.1).

Areas of* medium bone density (medium nB.Dn)*, that is, callus areas comprising 33-66% new bone, were more frequent than low bone density areas in all groups. In the* 0.0 mm group*, the* 0.2 mm group* and the* 0.4 mm group* medium bone density areas were very similar with 61.4 mm^2^ (SD 24.9), 65.1 mm^2^ (SD 19.0), and 71.4 mm^2^ (SD 45.1), respectively. Largest* medium nB.Dn* areas were found in the* 0.8 mm group* (100.5 mm^2^, SD 62.9).* Medium nB.Dn* was the commonest density in the control group averaging 81.3 mm^2^ (SD 37.8).

Areas of* high bone density (high nB.Dn),* that is, very compact callus areas comprising more than 66% newly formed bone, were predominant in all treatment groups. In the* 0.0 mm group*, the* 0.2 mm group*, and the* 0.8 mm group high nB.Dn* areas were very similar, averaging 129.2 mm^2^ (SD 13.6), 122.1 mm^2^ (SD 27.3), and 130.8 mm^2^ (SD 60.4), respectively. Largest high bone density areas were measured in the* 0.4 mm group* (164.3 mm^2^, SD 59.0). In the control group* high nB.Dn* areas were smallest (73.1 mm^2^, SD 27.6).

When looking at the ratio of low, medium, and high density areas within the callus, treatment groups showed a very similar proportional distribution: a relatively small area of* low nB.Dn* (5-10%), approximately 30% of* medium nB.Dn*, and 50-65% of* high nB.Dn*. However, in the control group a shift to less dense bone was observed: the upward tendency observed in the treatment groups levelled off at the medium density level in the control group (approximately 45% are* medium nB.Dn*). Areas with highly dense bone were less common (approximately 42%) as compared to the treatment groups.

Due to the small sample size the difference failed to reach statistical significance between the groups (p=0.556). No difference between the anterior and the posterior side of the callus concerning the proportion of low, medium, and high bone density areas was detected (p=0.371).

### 3.8. Regional Distribution of Different Bone Density Areas

The distribution of areas with different bone density over the total callus area was very similar between the groups and showed a consistent inherent pattern (Figures 3(a)–3(e)).

Areas of highly dense bone accounted for the majority of the total callus area and were predominantly located adjacent to the periosteal and endosteal surfaces of the tibial diaphysis and in the periphery of the periosteal callus. This finding was regardless of whether the gap was already bridged periosteally, endosteally, or intercortically. In specimens with an osseous bridged gap, highly dense bone was also found in the intercortical site and the endosteal compartment adjacent to the former gap.

Areas of medium dense bone most frequently occurred in specimens with a less developed osseous bridge in the intercortical region, that is, between the proximal and distal cortices. Independent of the grade of intercortical bridging, medium dense bone was found to be the predominant density bridging the anterior and the posterior endosteal surface within the marrow space. In addition, medium bone density areas were present at the outer margin of the periosteal callus at the level of osteotomy gap, and rarely between the cortical fragments. If cartilage was present (mainly in the periosteal gap), low and medium dense bone areas were surrounding cartilage remnants. Bone was observed to be denser with increasing distance to cartilage.

In all groups some individuals showed mid-sized to large areas of fibrous tissues within the callus.

## 4. Discussion

In this present study, a well-established delayed fracture healing model in the sheep [25] was used to analyse the effect of cyclic axial stimulation on the size of callus tissues and the spatial distribution of newly formed bone within the callus in terms of three density classes. A slight tendency towards an increase in size of total Cl.Ar, nB.Ar, and Cg.Ar was detected with rising displacement amplitudes. In addition, a side-specific difference of nB.Ar and Cg.Ar was identified between the anterior and the posterior callus side in all groups independent of treatment. Regarding the quality of callus, areas of very compact bone were predominant in the treatment groups whereas in the control group a slight shift to more porous bone was observed. Statistical power was very low due to the high complication rate in the control group.

### 4.1. Quantity of Callus Tissues

Our findings obtained by detailed semiautomatic histomorphometry are in line with other studies performed in sheep reporting increased new bone area in dynamically stabilised osteotomy gaps compared to more rigidly-stabilised gaps measured by various methods [9, 11, 15, 31]. A recent *μ*CT study of Tufekci et al. [19] demonstrated that 1 mm compressive IFMs, that were applied within the first 3 weeks, result in significantly increased bone volume compared to osteotomy gaps that were completely isolated from functional loading and IFM.

Considering the previous studies and the present results, displacement amplitudes in the range of 0.5-1 mm appear to have a potent stimulatory effect on bone healing in terms of increased callus formation. However, the timeframe of stimulation seems to be critical since bone is assumed to respond differently to mechanical strains depending on the stage of the bone healing process [12, 19, 23]. Recommendations concerning the optimal point in time for mechanical stimulation/dynamisation are conflicting [19, 21, 23, 24, 37]: Tufekci et al. demonstrated that the early stimulation within the first 3 weeks (starting 5 days after surgery) resulted in increased callus bone volume and torsional strength after 9 weeks in comparison with ongoing stimulation with decreasing displacement amplitudes. This so-termed “inverse stimulation” started with displacement amplitudes of 1 mm; stimulation was then continuously reduced in 0.25 mm increments until it was stopped after six weeks [19]. The authors demonstrated that, during the later stage of bone healing, mechanical stimulation is not needed. By contrast, it rather tends to compromise healing compared with early stimulation [19].

This is in clear contrast to Claes et al. who demonstrated superior healing with primary rigid (for at least 3 weeks) and subsequent flexible stabilisation of osteotomised femora of rats [23]. Compared to the constant flexible fixation group, callus area was decreased and bending rigidity was increased after 5 weeks. Late dynamisation was hypothesised to accelerate bone remodeling and consolidation of the callus [23]. In the current study, cyclic axial compressive dynamisation was also started comparatively late (12 days after surgery) and continued until 6 weeks postoperatively which is in between the dynamisation protocols of Tufekci et al. [19] and Claes et al. [23]. After 14 days, Epari et al. reported that periosteal woven bone formation is just about to start while soft callus composed of fibrous tissues is still present [20]. According to this, the present study reflects the effect of dynamisation on the beginning of hard callus formation.

The diverging results between these studies concerning the timing of dynamisation might be attributable to differences in the studied animal model, the gap size, the type of mechanical stimulation/dynamisation, the force and displacement amplitude of IFMs via loading or of external dynamisation, and the treatment duration, respectively. A standardised comparison of these dynamisation protocols is needed.

### 4.2. Quality of Callus Tissues

Since the quantity of the callus alone is not a useful predictor for mechanical stability of bone healing [9, 23, 26, 28], a second focus of this present study was set on a parameter of callus quality: the “density” or “compactness” of newly formed bone tissue or, in other words, the porosity of the calcified callus. In fact, mechanical axial dynamisation might have a slight effect on the density of newly formed callus: in the treatment groups, areas of high bone density were predominant whereas in the control group a slight shift to less dense bone was observed. The distribution of areas with different bone densities did not show an anterior- /posterior-specific pattern. Further studies with a larger study sample are needed to verify the observed tendency of this study. In addition, it has to be elucidated how the local distribution of areas with different compactness is correlated with bone strength and thus the resistance to refracture.

To our knowledge only a few studies were dealing with callus density. In these cases, density was defined as bone mineral density (BMD) of the callus or as bone area or bone volume per tissue area (BV/TV) which does not allow us to draw any conclusions about the distribution of newly formed bone within the callus and its actual porosity [20, 27, 33, 38, 39]. In this present study, we established a method to quantitatively describe the density or porosity of callus areas and the spatial distribution of new bone tissue within the callus. As there is no valid definition in the literature, we split densities into thirds of < 33% (low), 33-66% (medium), and > 66% bone tissue per callus area (high bone density). Despite the fact that the classification is not based on existing hard data, it provides interesting quantitative information about the distribution of bone tissue within the calcified callus that was qualitatively described in previous studies [20].

When interpreting the density or “compactness” of the callus, the stage of fracture healing and also the region (periosteal, endosteal, or intracortical) has to be considered. In this current study, moderate resorptive activity was predominantly present at the periosteal surface of the callus in all groups indicating that the ignition of external callus remodeling was rather at the same level in all groups. Accordingly, the lower bone density and also the lower nBV/TV in the control group do not seem to be the result of advanced resorption due to remodeling. From a theoretical point of view, they might rather be interpreted as a reduced need of bone mass in this area. In fact, the control group was equipped with two external fixators which provided higher stability. Most of the mechanical strains, which, under physiological conditions, are sustained by the bone itself, were now carried by the fixators. In consequence, in the control group, bone was subjected to reduced stresses when compared to the treatment groups. In other words, bone might have been understimulated [19] or stress shielded [40, 41]. Consequently, it might have simply been unnecessary for bone to invest in the formation of a larger and denser callus because stability was already provided. New bone might have only been formed to such an extent that stability is warranted in conjunction with the fixator. The mechanical phenomenon of “understimulation” or “stress shielding” of bone which occurs in areas where stability is already provided by an implant or fixator is mainly described for joint prosthesis in the hip or the knee [40, 42]. However, “stress shielding” was recently also described as being a potential risk of stiff osteotomy or fracture fixation allowing insufficient axial compression via external fixators [43–46]. Studies dealing with the effect of stress shielding mainly report bone resorption of bone areas adjacent to prosthesis or implants that are no longer subjected to mechanical strains due to changes in load transfer after implant integration [42]. Since the local strain distribution at an osteotomy site is assumed to provide mechanobiological signals that lead to a specific pattern of tissue distribution [47] it is conceivable that stress shielding also might have a direct effect on the bone formation process in osteotomy and fracture healing resulting in a reduced callus area and callus “density.”

In order to draw conclusions about the potential role of stress shielding in the context of external fixation of osteotomies, long-term studies are required analysing the course of bone formation from its beginning up to that point when an equilibrium between bone formation and bone resorption is achieved. The bone defect model established by Tufekci et al. (2018) which isolates an osteotomy gap from any kind of IFM might be a good tool to investigate the effect of “understimulation” on bone formation and callus density over the course of time.

### 4.3. Anterior/Posterior Difference in Callus Formation

The third focus of this present study was put on the regional distribution of newly formed tissues between the anterior and the posterior side of the callus. Several studies reported a side-dependent difference in callus formation between the medial and the lateral callus side when the osteotomies were stabilised with rigid or flexible fixators mounted on the medial aspect of the tibia [12, 20]. In this present study, we examined if differences exist also in the anterior-posterior plane when the fixator is mounted on the lateral side of the tibia. In fact, anterior nB.Ar was almost twice as high as posterior nB.Ar in all groups. The reasons for this obvious bias towards one side are not completely understood. When looking for potential explanations of this side-difference we came across the stiffness properties of the unilateral external fixator. In fact, 4-point bending stiffness of the unilateral fixator was significantly reduced in the anterior-posterior direction (31 N/mm) compared to the mediolateral direction (167 N/mm) in this present study as previously described by Wolf et al. [29]. This suggests the assumption that, in the treatment groups, interfragmentary movement in the anterior-posterior direction was possible during weight bearing at least to some extent. However, the same pattern of bone distribution was observed in the control group in which stiffness in the anterior-posterior direction was increased to 170 N/mm by the application of the second external fixator.

As reported by others, the orientation of an external unilateral fixator is potent to cause nonuniform interfragmentary movements and additional shear movements within the osteotomy gap [12, 15, 17, 48] which in turn might influence bone healing in one way. Beyond the mounting plane of the external fixators and the associated changes in shear forces, muscle strains, and gait patterns, also the greater soft tissue coverage at the anterior side and one-sided damage of blood vessels might contribute to differences in cell activation and thus in the temporal and spatial callus formation [12, 20, 49–51]. Further examinations are needed to determine if the observed anterior-posterior difference also exists in tibias being stabilised with medially mounted external fixators.

### 4.4. Limitations

Unfortunately, five sheep of the four treatment groups and five sheep of the control group had to be excluded from the study due to severe complications (Table 1). Pin track infections and fractures through the screw holes proximally and distally of the osteotomy gap occurred in more than 55% of the animals of the control group which led to a very small group size and weak power of our statistics.

A potential reason for the high drop-out rate in the control group might be the localisation and the increased number of screws that was necessary to fix the second external fixator [30]. The four additional screws undoubtedly resulted in an additional local tissue trauma and thus in an increased infection risk at the pin insertion sites. Also the anterolateral localisation of those screws might have been unfavourable due to its vicinity to muscle tissue. Weight bearing during walking leads to relative movements between the soft tissue and the screws, thus provoking muscle and soft tissue irritation, which is reported to be the most common reason for pin infections. [52] Similar complication rates were reported for studies in sheep and rabbits due to pin infections, pin loosening, or fractures [9, 53]. Also, in the clinical setting, pin site infections and fracture susceptibility of the screw holes are common complications [46, 54] but the better hygienic conditions facilitate prevention and healing in human patients.

Considering these complications and the high exclusion rate of animals it would have been very desirable to conduct additional animal experiments especially in order to increase the low number of control animals. As mentioned, the current study reevaluated sheep tibiae from an animal experiment performed about twenty years ago [29, 30]. We found that under these conditions it would not have been possible to perform a repetition of the surgery with the required degree of standardisation a posteriori. Therefore we decided not to supplement this current study with any new animal experimentation consciously accepting the low number of control animals.

Beyond the small group sizes, the main limitation of this study is that callus formation was analysed only after six weeks which makes it impossible to draw conclusions about potential differences between the groups in the early healing period. It remains open if the observed slight differences in callus densities are related to the timing of dynamisation. To analyse the progress of callus formation multiple time points should be considered.

## 5. Conclusion

In brief, axial dynamisation tended to slightly increase the volume of the newly formed callus. Besides, mechanical stimulation might have a slight effect on the density or compactness of the calcified callus. It could be demonstrated that the established method to assess the density or porosity of callus areas is a useful tool to quantitatively determine the spatial distribution of new bone tissue within the callus. The application of this method in combination with biomechanical testing might reveal interesting relations between tissue distribution of healing bone and bone strength that, with the traditional histomorphometric parameters BV/TV or B.Ar, cannot be identified.

In addition to the medial/lateral differences in new bone volume described in the literature, this study demonstrated that there exists also a difference between the anterior and posterior side. If this side difference also persists if the mounting plane of the external fixators is changed is issue of further analysis.

## Figures and Tables

**Table 1:** Treatment groups and stimulation regimen.

***Group***	***Evaluated Animals (n)***	***Excluded Animals (n)***	***Fixation Device***	***Mechanical Stimulation***
**0.0 mm group**	6	0	unilateral external fixator	no stimulation
**0.2 mm group**	6	1	unilateral external fixator	1200 cycles/day with 1 Hz, amplitude 0.2 mm
**0.4 mm group**	7	2	unilateral external fixator	1200 cycles/day with 1 Hz, amplitude 0.4 mm
**0.8 mm group**	8	2	unilateral external fixator	1200 cycles/day with 1 Hz, amplitude 0.8 mm
**control group**	4	5	unilateral external fixator AND unilateral monotube external fixator	no stimulation

***Total***	**31**	10		

**Table 2:** Mechanical properties of external fixator configurations [30].

	***One-plane fixation*** (Stimulation groups)	***Two-plane fixation*** (Control group)
	***unilateral external fixator***	***unilateral external fixator*** * AND * ***unilateral monotube ext. fixator***
**Axial stiffness**	183 N/mm	388 N/mm
**Torsional stiffness**	2.5 Nm/deg	2.5 Nm/deg
**Bending stiffness in mediolateral direction**	2Nm/deg (167 N/mm )	3 Nm/deg (250 N/mm*∗*)
**Bending stiffness in anterior-posterior direction**	31 N/mm	170 N/mm

*∗* Estimated value. Comparative data for bending stiffness in mediolateral direction given in N/mm is missing in Krischak et al. (2002)

**Table 3:** nB.Ar and Cg.Ar were assessed separately for the anterior and the posterior callus side. Anterior new bone formation was up to twice as high as posterior. Also cartilage area was significantly higher at the anterior callus side. Data are presented as mean values (mean), standard deviations (SD), median, minimum (Min), and maximum (Max) in mm.

	***Location***	***Treatment Group***	***Mean***	***SD***	***Median***	***Min***	***Max***
**nB.Ar**	**anterior**	0.0 mm	88.1	21.2	92.6	47.0	107.8
		0.2 mm	70.9	32.3	74.4	18.6	109.8
		0.4 mm	111.7	38.4	99.5	65.4	179.3
		0.8 mm	103.8	36.4	111.9	42.3	158.5
		Control	70.7	44.0	65.6	27.0	124.7
	**posterior**	0.0 mm	48.8	27.3	44.4	22.3	98.1
		0.2 mm	63.1	25.4	56.6	34.9	102.1
		0.4 mm	58.8	28.1	56.4	17.4	99.1
		0.8 mm	56.8	25.5	54.2	27.1	96.0
		Control	34.4	9.1	35.4	23.0	43.6
	**Total**	0.0 mm	136.9	16.0	146.4	116.0	147.9
		0.2 mm	134.0	20.4	131.3	109.2	156.4
		0.4 mm	170.5	53.1	164.5	106.1	260.8
		0.8 mm	160.6	55.8	158.2	85.5	230.7
		Control	105.1	43.6	98.6	67.1	156.2

**Cg.Ar **	**anterior**	0.0 mm	5.2	4.4	4.3	1.0	10.4
		0.2 mm	5.9	4.6	7.1	0.0	11.0
		0.4 mm	8.7	9.3	7.6	0.3	25.1
		0.8 mm	5.6	3.0	5.4	1.9	11.7
		Control	0.3	0.3	0.2	0.0	0.7
	**posterior**	0.0 mm	1.3	2.4	0.2	0.0	6.0
		0.2 mm	3.5	1.9	3.9	1.0	5.6
		0.4 mm	2.4	3.3	0.4	0.0	8.5
		0.8 mm	2.4	2.1	1.8	0.4	6.6
		Control	0.0	0.0	0.0	0.0	0.0
	**Total**	0.0 mm	6.6	4.5	7.4	1.0	11.9
		0.2 mm	9.4	5.7	10.4	2.0	16.7
		0.4 mm	11.1	10.1	9.3	0.3	30.2
		0.8 mm	7.9	4.4	8.1	2.4	15.2
		Control	0.3	0.3	0.2	0.0	0.7

**Table 4:** nBV/TV and CgV/TV were assessed separately for the anterior and the posterior callus side. Although the absolute area of callus tissues is significantly different between the anterior and the posterior side, the relative amount of new bone within the anterior and the posterior callus area (nBV/TV) is very similar. Data are presented as mean values (mean), standard deviations (SD), median, minimum (Min), and maximum (Max) in %.

	***Location***	***Treatment Group***	***Mean***	***SD***	***Median***	***Min***	***Max***
**nB.Ar**	**anterior**	0.0 mm	67.6	5.0	67.8	59.4	74.6
		0.2 mm	64.0	11.4	62.9	50.0	82.7
		0.4 mm	66.4	9.9	67.1	50.8	78.8
		0.8 mm	60.2	11.2	61.1	37.3	74.9
		Control	59.7	2.7	59.1	57.6	63.3
	**posterior**	0.0 mm	64.4	7.1	63.8	53.2	72.3
		0.2 mm	62.5	7.4	62.3	54.0	75.1
		0.4 mm	65.8	8.8	65.5	57.5	77.8
		0.8 mm	61.9	12.9	64.9	32.4	75.3
		Control	55.0	8.4	57.3	42.9	62.5
	**Total**	0.0 mm	66.4	4.2	65.1	62.7	74.0
		0.2 mm	61.6	7.1	62.0	51.2	72.6
		0.4 mm	66.0	8.9	63.3	52.8	78.2
		0.8 mm	60.6	12.0	62.3	34.6	75.0
		Control	58.6	3.6	59.4	53.9	61.7

**Cg.Ar **	**anterior**	0.0 mm	3.7	2.6	3.6	0.8	6.5
		0.2 mm	3.9	2.7	4.8	0.0	6.3
		0.4 mm	4.3	4.3	3.3	0.3	10.9
		0.8 mm	3.4	1.8	3.2	1.3	6.3
		Control	0.2	0.3	0.2	0.0	0.5
	**posterior**	0.0 mm	1.1	1.6	0.3	0.0	3.8
		0.2 mm	3.7	2.3	3.1	1.1	6.9
		0.4 mm	2.0	2.3	1.0	0.0	5.2
		0.8 mm	2.8	2.6	2.2	0.4	7.8
		Control	0.0	0.0	0.0	0.0	0.0
	**Total**	0.0 mm	3.0	1.9	3.3	0.6	5.3
		0.2 mm	4.0	2.1	4.6	1.2	6.5
		0.4 mm	3.8	3.2	2.9	0.2	9.2
		0.8 mm	3.2	1.9	3.0	1.0	5.5
		Control	0.2	0.2	0.1	0.0	0.3

## Data Availability

The data used to support the findings of this study are available from the corresponding author upon request.
